# Commissioning and validation of the Elekta One GPUMCD algorithm for an Elekta VersaHD linear accelerator

**DOI:** 10.1002/acm2.70540

**Published:** 2026-03-20

**Authors:** Samuel D. Rusu, Blake R. Smith, Grace H. Hutchinson, Jun Hsuang Jen, Daniel E. Hyer

**Affiliations:** ^1^ Department of Radiation Oncology University of Iowa Hospitals and Clinics Iowa City Iowa USA

**Keywords:** Dose validation, GPUMCD, Monte Carlo

## Abstract

**Purpose:**

The purpose of this work was to commission and validate GPUMCD, a GPU‐accelerated Monte Carlo dose calculation engine for c‐arm Elekta linear accelerators (linac). This algorithm was recently released for clinical implementation in the Elekta One Treatment Planning System (v6.2.3, EOP).

**Methods:**

A GPUMCD beam model was generated for all photon energies of a VersaHD linac (6X, 6FFF, 10X, 10FFF, 18X). A validated version of the Monaco Commissioning Utility was used to compare calculated percent depth dose (PDD) profiles as well as lateral profiles for open fields against measurements. An adapted MPPG 5.b methodology was used to verify point‐doses and 3D dose distributions in homogeneous and heterogeneous media using the ArcCheck, solid water, the CIRS ZEUS phantom, and the IROC HN and spine phantoms.

**Results:**

The average agreement between measured and calculated PDDs and beam profiles using a local 2% dose difference (DD) in the high dose region for fields greater than 5 × 5 cm^2^ was 98.4% ± 2.3% for all energies. Using a 2% DD and 2 mm distance‐to‐agreement (DTA) gamma criteria for all fields using a 5% dose threshold yielded an agreement of 99.9% ± 0.5%. For open fields, GPUMCD reduced the calculation time by 93% as compared to X‐ray voxel Monte Carlo (XVMC) using the same hardware. All MPPG 5.b. recommended testing was within the suggested tolerance limits. All plan measurements passed at the recommended gamma criteria. GPUMCD heterogeneity agreement and point dose measurements were found to agree within 3%.

**Conclusion:**

The GPUMCD algorithm in EOP was successfully tested and commissioned for clinical use for the VersaHD linac.

## 1 INTRODUCTION

Monte Carlo (MC) methods are considered the gold standard for dosimetric computations in radiotherapy, and computational power and speed have historically limited them from routine clinical use.^1^ Improved accessibility to powerful computational architecture has made the commercial use of MC based algorithms for daily and adaptive plans possible, including the incorporation of GPU‐accelerated dose calculation.^2^, ^3^, ^4^ Recently, Elekta released the GPU‐based MC dose calculation algorithm (GPUMCD) to be used clinically in the Elekta One Treatment Planning System (EOP) (v.6.2.3) treatment planning system (TPS) for the Elekta VersaHD linac in March of 2025.

GPUMCD is a MC radiation transport code that was designed to perform rapid dose calculations using GPUs. While XVMC represents beam modifiers as transmission filters with constant‐Z planes that allow particles to pass, be blocked, or have their statistical weights adjusted, GPUMCD models beam modifiers as full three‐dimensional material objects, enabling incident particles to be transported through them using MC methods.^5^, ^6^ GPUMCD also uses particle splitting and Woodcock ray tracing for variance reduction techniques while XVMC uses interaction forcing, electron history repetition, and Russian Roulette.^5^, ^6^ As a result, new clinical beam models that are different from those used for XVMC are needed for GPUMCD.

GPUMCD has been compared to EGSnrc dosimetrically in 2010 and was found to agree better than 98% (2%‐2 mm gamma) for significant voxels and be three orders of magnitude faster than EGSnrc.^1^ Due to its accuracy and speed, Elekta has implemented GPUMCD into EOP, for photons only, and it has been used clinically for the Unity MRI linear accelerator since 2019. GPUMCD was shown to agree within 2.5%/2.5 mm gamma criteria for film measurements within the tumor‐lung interface and within a 2%/2 mm gamma criteria near bone heterogeneities for 6MV on an Elekta Agility linac, which shows improved agreement with film measurements at these interfaces as compared to XVMC.^7^ Given the scarcity of literature that exists for the commissioning of the GPUMCD for the Elekta VersaHD linacs, it was the aim of this work to share our initial clinical experience of implementing GPUMCD with new beam models in EOP for the Elekta VersaHD linac at our institution and to enable a wider application of this algorithm and TPS for other clinics.

## 2 METHODS

GPUMCD models were generated by the manufacturer for 6MV, 6FFF (flattening filter free), 10MV, 10FFF, and 18MV from the collected water scan data and an adapted MPPG 5.b. methodology was used for the commissioning and validation of the GPUMCD algorithm.^8^

### 2.1 Beam data collection

Beam data was scanned at 90 cm source‐to‐surface distance (SSD) in a PTW MP3‐m water tank using the PTW's MEPHYSTO mc^2^ Navigation software for scanning and post‐processing following the TG‐106 protocol.^1^ A PTW TW60019 microDiamond was used for measuring PDDs for field sizes of 7 × 7 cm^2^ and smaller. The microDiamond along with the SNC Edge detector were used for lateral profile measurements of fields smaller than 7 × 7 cm^2^ and were averaged to determine output factors. All other scan data was measured with a PTW 31010 chamber (0.125 cm^3^ active volume).

### 2.2 Open‐field algorithm timing comparison

To investigate the time difference and time reduction observed between XVMC and GPUMCD open fields ranging from 2 × 2 cm^2^ to 40 × 40 cm^2^, as reported by EOP, were calculated with a 0.2 cm dose grid and a 0.5% uncertainty on the same hardware.^9^

#### 2.2.1 Open‐field dosimetric verification and profile comparison

A 50 × 50 × 50 cm^3^ cube was used for open‐field beam calculations in EOP. All calculations were recorded as dose to water and an absolute dose verification was performed at three points of interest: a 10 × 10 cm^2^ field at 90 cm SSD and 10 cm depth in addition to a 10 × 10 cm^2^ field at 100 cm SSD for depths at dmax and 10 cm. Individual dose calibrations were applied to the beam model to achieve agreement within 0.5%.

For fields sizes less than 5 × 5 cm^2^, a 0.1 cm dose grid with 0.2% uncertainty was used to calculate the dose. For fields greater than 5 × 5 cm^2^, a 0.2 cm dose grid with 0.3% uncertainty was used to calculate the dose. The dose per beam was DICOM exported to be analyzed using the Monaco Commissioning Utility v 1.0.0.296 (MCU) and compared to the in‐water scans. A local 2% absolute dose difference (DD) was used to evaluate percent depth dose (PDD) agreement up to a depth of 29.5 cm. In the high dose region (> 80% of the central axis dose), a local 2% absolute DD was used to evaluate the lateral profiles for field sizes greater than 5 × 5 cm^2^ while a 2% DD and 1 mm DTA local gamma criteria was used for fields smaller than 5 × 5 cm^2^. For completeness, a global 2% absolute DD and 2 mm distance‐to‐agreement (DTA) gamma criteria with a 5% dose threshold were used to evaluate agreement for all profiles globally.

#### 2.2.2 Point dose, output factor validation, and inhomogeneity validation

Output factors were calculated at a depth of 10 cm and compared against measured values. The measured output factors were used in combination with the profile data in MCU for absolute dose profile comparison. Point dose calculations were calculated to water with a 2 mm dose grid, 0.2% uncertainty, and phantom lookup table and measured with a 0.125 cc PTW 31010 cross‐calibrated chamber ion chamber unless otherwise specified.

**FIGURE 1:**
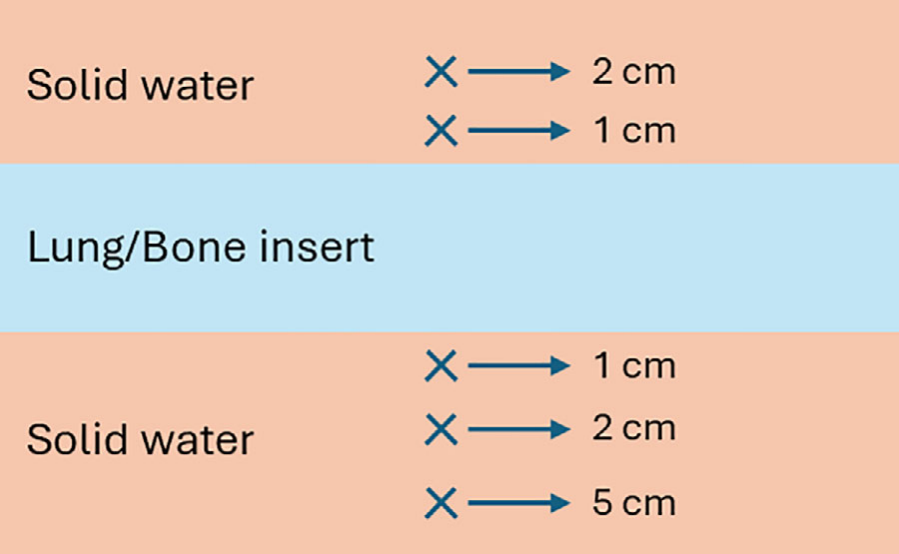
Inhomogeneity AP measurement setup with the X designating measurement locations.

**FIGURE 2:**
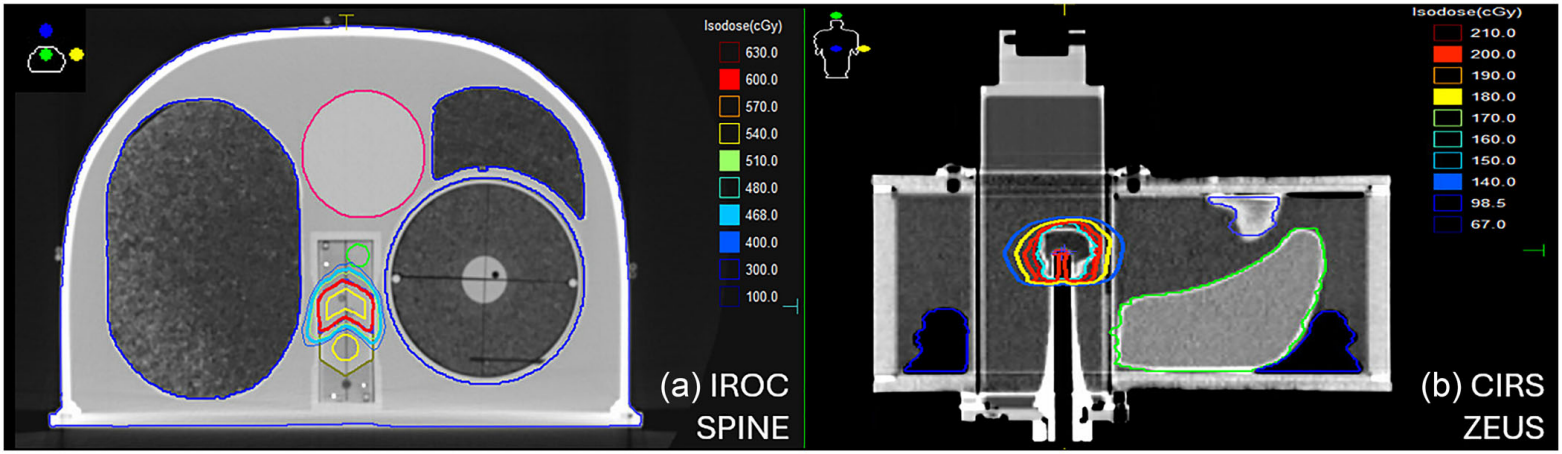
Anthropomorphic phantoms used to test dose calculation accuracy in heterogeneous media. (a) The IROC spine phantom with a target region in a simulated spine with tissue representative inserts for lungs and bone. (b) Point dose measurement were performed in the CIRS ZEUS phantom for a simulated abdominal anatomy.

The agreement of the GPUMCD dose calculation algorithm in regions adjacent to low‐ and high‐density heterogeneities were evaluated in accordance with the guidelines of MPPG 5.b. In both cases, 200 MU was delivered to a 10 × 10 cm^2^ field size with a 100 cm SSD for commissioned photon energies. Dose accuracy near low‐density interfaces was assessed by calculating the dose delivered proximal and distal to a 7.5 cm thick foam insert position between slabs of solid water. A similar methodology was applied to evaluate dose accuracy near high‐density interfaces, with the foam insert replaced with 3.5 cm of bone‐equivalent phantom slab. Measured and calculated doses were compared at positions located 2 and 1 cm proximal to, as well as 1 cm, 2 cm, and 5 cm distal to the heterogeneity interface as seen in Figure 1. Heterogeneity testing using anthropomorphic phantoms were evaluated using the CIRS phantom and externally validated using the IROC phantoms as seen in Figure 2. This is discussed in detail in sections 2.3.3 and 2.3.4.

### 2.3 Plan validation

The MPPG 5.b. methodology was adapted, and plans were generated for MPPG 5.b. sections 5.4–5.8 for all energies. The 3D dose distribution and the point dose agreement were compared using the ArcCheck and an ion chamber.

#### 2.3.1 ArcCheck electron density (ED) optimization

The Sun Nuclear manual was followed to optimize the ArcCheck ED in EOP using SNC Patient (v.8.5.0.110). An array and dose calibration were performed before optimization occurred. A virtual phantom of the ArcCheck was loaded into EOP and the 2 cm rods were set to a relative ED of 1.40. A plan was generated in EOP and delivered to the ArcCheck using a 10 × 10 cm^2^ field size, 100 cm source to axis (SAD) distance, and 200 MU for all energies. The entrance to exit diode ratio was calculated and the measured values were averaged over two detectors for both the entrance and exit values, as specified in the manual. The measured and calculated entrance and exit ratios were then evaluated with a 1% agreement desired. Two fields, at gantry 90 and 270, were also measured for local DD comparison. The local gamma analysis (2%, 2 mm, 10% dose threshold) as defined in SNC Patient was evaluated, and the ArcCheck relative ED was then iteratively adjusted until the best agreement was found across all energies. A point dose comparison in the center of the ArcCheck for all energies for the gantry angles measured was also performed to evaluate the agreement of the optimized relative ED value.

#### 2.3.2 Couch modeling

The Hexapod evo RT couch top and the Civco Universal Couchtop (REF: MTIL6040) were modeled as a single couch model in EOP. Two dynamic conformal arcs (20 × 20 cm^2^) for each energy were calculated in EOP so that an effective 180‐degree arc going through the couch would be delivered to the ArcCheck. These plans were measured with a cross calibrated 0.125 cm^3^ ion chamber point measurement in the center of the ArcCheck. An AP/PA ion chamber measurement was also taken in the center of the ArcCheck. The optimal couch relative ED parameters were iteratively adjusted to get the best agreement across all measurements.

#### 2.3.3 IMRT/VMAT plan validation

The TG‐119 provided C‐shape and HN CT and structure sets were imported into EOP and were used to commission IMRT and VMAT treatments. Test plans for 6X, 6FFF, 10X, and 10FFF (total 24 plans) were generated to meet or exceed the mean and standard deviation of both the hard C and easy C objectives as well as the HN objectives for the respective structure sets.^10^ The IMRT plans used nine equally spaced beams with a minimum segment width of 0.5 cm, minimum segment areas of 2 cm^2^, and a max of 128 segments per plan for both DMLC and Step and Shoot IMRT plans. Easy C VMAT plans were generated using two full arcs with one sweep using 30‐degree arc increments with a zero‐collimator rotation. HN VMAT plans were generated using one arc with two sweeps using 30‐degree arc increments with a zero‐collimator rotation. Hard C VMAT plans were generated using 2–3 arcs, 10–30 arc increments, and non‐zero collimator rotations. All VMAT plans were limited to 120 control points per arc. All plans were generated using a 2 Gy per fraction scheme with a dose calculation grid of 2 mm with 1% uncertainty per calculation.

Previously treated sample patient VMAT plans for each energy were imported and recalculated. The selected head and neck and prostate plans followed a standard fractionation scheme of 2 Gy per fraction. The lung and pelvic node followed received 54 and 24 Gy in 3 fractions respectively and were planned to achieve a heterogeneous target dose and sharp dose falloff. The lung plan consisted of two partial arcs to avoid the contralateral lung, and the pelvic node had one full arc.

All plans were mapped as QA plans on the ED‐optimized ArcCheck including the optimized couch model. Dose was calculated to water using the phantom lookup table with a 2 mm dose grid with 1% uncertainty. Gamma analysis was performed following the recommendations of TG‐218 of 3%/2 mm and additionally evaluated using MPPG 5.b. recommendation of 2%/2 mm passing criteria using absolute dose, global normalization, and a low‐dose threshold of 10%.

Point dose measurements for IMRT plans were measured using the methodology described in TG‐119 and calculated with the optimized couch model. The generated IMRT plans were mapped to a 15 cm slab of solid water and calculated for point dose comparison. Point dose measurements for VMAT plans were measured at different locations in the ArcCheck. This included isocenter and at chosen points in high‐dose, low‐gradient regions or low‐dose regions within the ArcCheck. Comparative dose values for each measurement were calculated from the mean dose within a volume equal to the size of the sensitive ion chamber volume.

End‐to‐end measurements were performed for the subset of VMAT beam energies using the CIRS ZEUS (008z‐b‐cv57‐9) phantom. Pre‐treatment quality assurance was performed following the same methodology previously described. The phantom was aligned using CBCT image guidance and delivered under clinical conditions. Point dose measurements in the target were obtained and compared to the TPS predicted point dose using the patient lookup table.

#### 2.3.4 IROC external validation

Treatment plans for the spine and head and neck IROC phantoms were generated and delivered to the phantoms following the IROC guidelines using in‐house immobilization. These phantoms were then sent to IROC to externally validate the dose calculation algorithm and calculated dose.

## 3 RESULTS

### 3.1 Open‐field algorithm timing results

Table 1 shows the time difference and percent time reduction for a sample of open‐field sizes. GPUMCD provided an average calculation time reduction of 748.4s for open fields, which is a 92.6%‐time reduction when comparing XVMC and GPUMCD.

**TABLE 1:** Time difference in seconds and percent reduction between XVMC and GPUMCD for field sizes (cm) (0.2 cm dose grid, 0.5% uncertainty).

Field size	XVMC	GPUMCD	Time difference	% reduction
2 × 2	83.9	1.3	82.6	98
5 × 5	163.5	6.6	156.9	96
10 × 10	355.7	25.1	330.5	93
20 × 20	1072.5	111.0	961.5	90
40 × 40	2568.6	358.1	2210.6	86
		**Average**	748.4	92.6

#### 3.1.1 Open‐field dosimetric verification and profile validation

The absolute dose verification at the three points of interest was found to agree within 0.5%. All profiles agreed well for the models generated. Specifically, for 6X, 6FFF, 10X, 10FFF, and 18X the PDDs and profiles on average using a local DD agreed to 98.1% ± 2.4%, 99.4% ± 1.5%, 98.1% ± 1.2%, 98.8% ± 3.1%, 98.0% ± 2.2% respectively. The average gamma pass rate was 99.9% ± 0.4%, 100.0% ± 0.2%, 99.9% ± 0.4%, 100.0% ± 0.0%, and 99.8% ± 0.9%. A subset of PDD and profile comparison between GPUMCD (green) and measured data (blue) of filtered beams and filter free beams for small and large fields is seen in Figure 3 (a) and (b) respectively. These graphs were generated using the MCU and the red lines indicate the gamma (2%/2 mm) or local dose deviation in the region of interest. The output factor normalized dose corresponds to the scaled PDD based on the output factor relative to the dose under reference condition.

FIGURE 3(a) Subset of PDD and profile comparisons between GPUMCD (green) and measured data (blue) of filtered beams for small and large fields. (b) Subset of PDD and profile comparisons between GPUMCD (green) and measured data (blue) of FFF beams for small and large fields.

**Figure:**
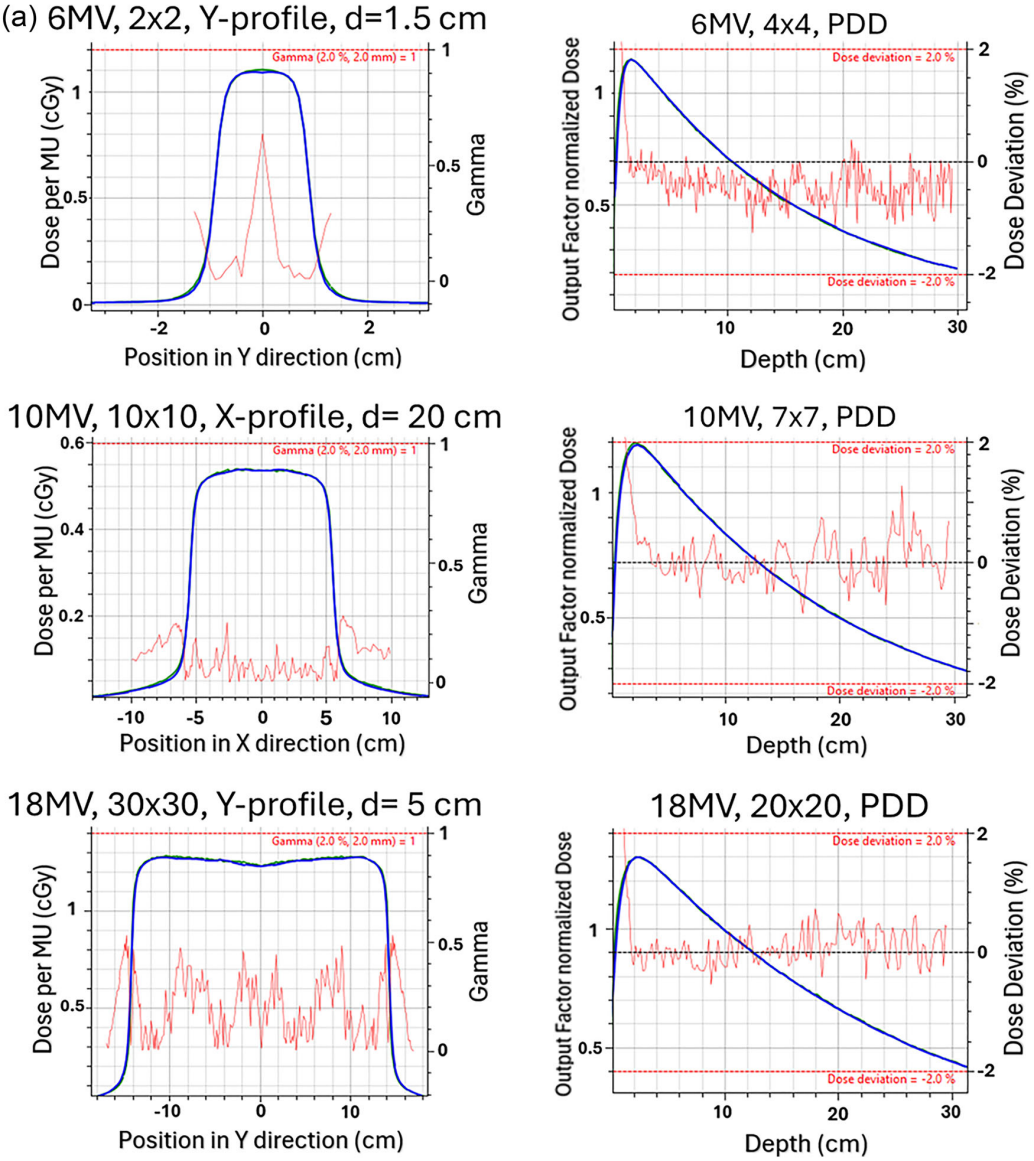

**Figure:**
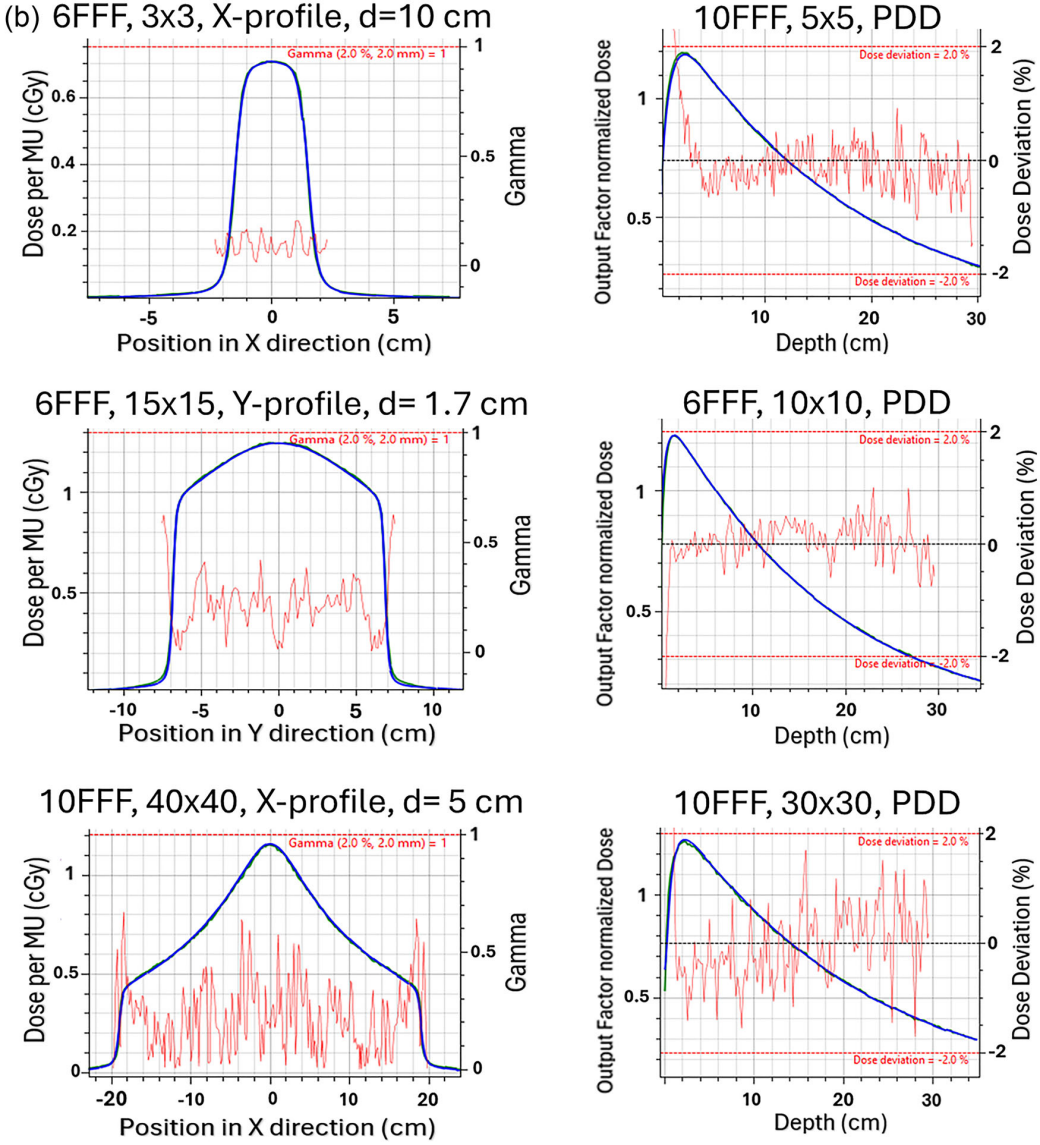

#### 3.1.2 Point dose, output factor validation, and inhomogeneity validation results

All point doses and output factors for all energies agreed within 2%. A sample subset of output factor agreement is presented in Table 2.

**TABLE 2:** A selection of output factor agreement for small and large field sizes (cm).

Energy	Field size (cm^2^)	% (Meas‐Calc)/Calc
6X	2 × 2	0.50
	40 × 40	−0.24
6FFF	3 × 3	0.25
	30 × 30	−0.29
10X	4 × 4	0.84
	20 × 20	0.19
10FFF	5 × 5	−0.04
	15 × 15	−0.10
18X	7 × 7	−0.11
	5 × 40	−0.25

**FIGURE 4:**
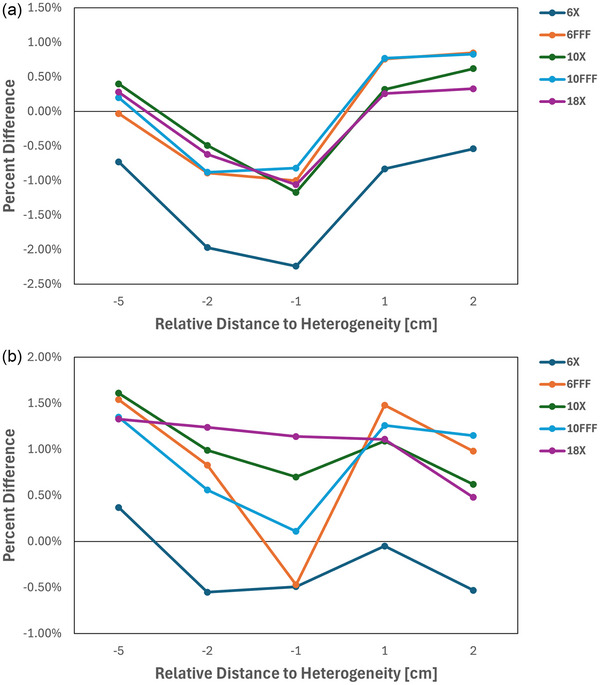
(a) High‐density inhomogeneity measurements compared to GPUMCD calculation with the distance specified relative to the center of the inhomogeneity layer, a negative number denotes posterior measurements, and a positive number denotes anterior measurements. (b) Low‐density inhomogeneity measurement compared to GPUMCD calculation with the distance specified relative to the center of the inhomogeneity layer, a negative number denotes posterior measurements, and a positive number denotes anterior measurements.

All measured and calculated doses were within 3% for both low‐ and high‐density heterogeneities. The largest discrepancy for the low‐density comparison was observed 5 cm below the heterogeneity interface at 10X energy, with a percent difference of 1.61%. For the high‐density scenario, the greatest discrepancy occurred 1 cm below the interface with an energy of 6X, with a percent difference of −2.24%. Results are summarized for low‐ and high‐density heterogeneities in Figure 4(a) and 4(b), respectively.

#### 3.1.3 ArcCheck ED optimization

An ED of 1.164 was the optimal value determined for ArcCheck measurements at our institution. At this density, the entry/exit ratio between measured and calculated for all the energies was found to be 0.06% ± 0.55% using EOP and 0.4% ± 0.8% using SNC Patient. The average local distance to agreement (2%, 2 mm, 10% dose threshold) as defined in SNC Patient for all measured fields (12 fields total) was found to be 95.2% ± 3.1%. The average percent difference of the point dose measurements for all fields were found to be 0.1% ± 0.2% for all energies. The beam energy of 18X was not used to optimize the ED value since it will not be used for VMAT deliveries.

#### 3.1.4 Couch Modeling

The optimized relative ED of the inside of the couch model was 0.02 and the outside of the couch was 0.2. These values resulted in the best local distance to agreement (2%, 2 mm, 10% dose threshold) across the two different couch models and point dose measurements within 1% of calculated values.

#### 3.1.5 IMRT/VMAT plan validation

Both ArcCheck measurements of generated plans and point dose measurements in high and low dose regions agreed well with the dose values calculated in EOP. Table 3 summarizes the ArcCheck measurements done for the generated TG‐119 IMRT/VMAT plans. Composite and per beam averages of the 3%/2 mm and 2%/2 mm gamma analysis are reported for each energy.

**TABLE 3:** TG‐119 IMRT/VMAT average percent gamma passing rates and standard deviation across all TG‐119 plans.

Energy	Composite gamma (3%/2 mm)	Per‐field gamma (3%/2 mm)	Composite gamma (2%/2 mm)	Per‐field gamma (2%/2 mm)
6 MV	98.0 ± 1.1	97.0 ± 2.2	96.0 ± 1.9	95.3 ± 3.1
6 FFF	97.8 ± 2.6	97.4 ± 2.5	95.8 ± 3.8	95.3 ± 3.7
10 MV	97.7 ± 1.6	97.4 ± 1.7	95.6 ± 2.1	95.5 ± 2.2
10 FFF	98.9 ± 1.1	98.1 ± 2.0	97.5 ± 1.9	97.1 ± 1.9

A summary of the TG‐119 IMRT/VMAT percent point DD is presented in Table 4. All measurements for high and low dose reasons agreed within 3%. The clinical plan measurement results are summarized in Table 5 and compared to XVMC for reference. An average 99.8% 3%/2 mm gamma with a 10% dose threshold on the ArcCheck was observed for the replanned clinical plan in EOP. The end‐to‐end point dose results all agreed within a 1%‐point dose measurement with an average 98.2% 3%/2 mm gamma with a 10% dose threshold on the ArcCheck. The actual differences observed per energy are found in Table 6.

**TABLE 4:** TG‐119 IMRT/VMAT percent point dose difference and standard deviation averaged across all TG‐119 plans.

Energy	IMRT		VMAT	
	High dose region	Low dose region	High dose region	Low dose region
6 MV	1.2 ± 1.6	1.5 ± 1.2	2.1 ± 0.8	0.9 ± 1.2
6 FFF	2.4 ± 0.1	1.9 ± 0.2	−0.9 ± 1.5	−0.3 ± 2.5
10 MV	1.1 ± 1.6	2.2 ± 0.5	0.8 ± 1.1	0.3 ± 1.8
10 FFF	0.7 ± 1.0	1.4 ± 1.1	−0.4 ± 1.8	0.8 ± 1.3

**TABLE 5:** Clinical plan percent gamma passing rates.

Energy	Plan	XVMC gamma (3%/2 mm)	GPUMCD gamma (3%/2 mm)
6 MV	HN	99.3	99.7
6 FFF	Left lung SBRT	100.0	100.0
10 MV	Prostate	90.9	99.4
10 FFF	Pelvic node	99.5	100.0

**TABLE 6:** End to end percent point dose difference and QA gamma percent passing rates.

Energy	Percent dose difference (%)	QA gamma (3%/2 mm)
6 MV	0.94	98.0
6 FFF	0.08	98.5
10 MV	0.87	96.7
10 FFF	−0.22	99.5

#### 3.1.6 IROC external validation results

**FIGURE 5:**
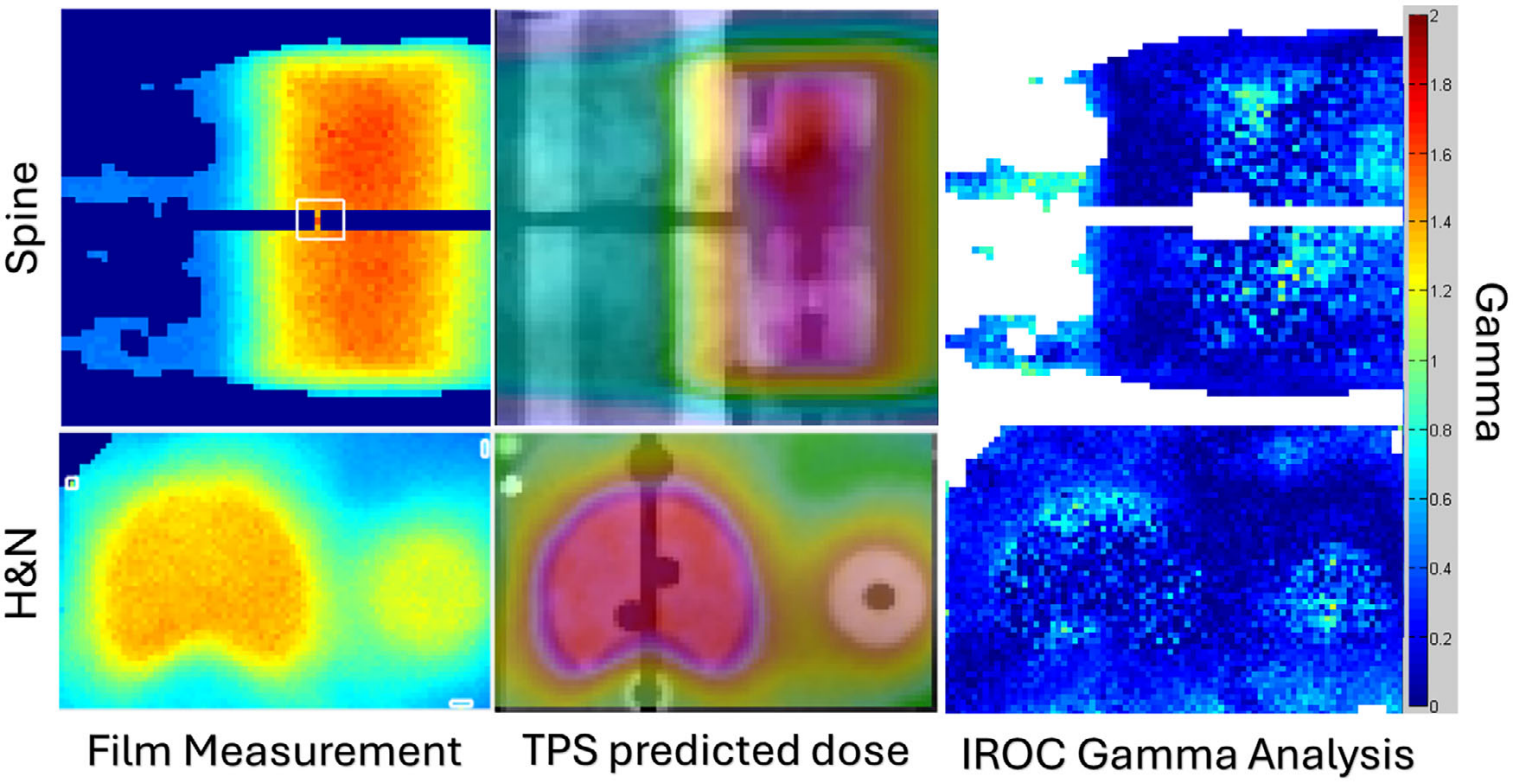
A comparison between film measurement, TPS predicted dose, and 2D gamma analysis reported from IROC for the spine and H&N phantoms.

The spine phantom met the criteria established by IROC and had an average Gamma index, which IROC defines as percentage of points meeting their gamma‐index criteria, of 98% both film planes. The average target TLD agreement was within 1% between the measured and predicted dose values with the worst agreement within 3%. The HN phantom met the criteria established by IROC and had an average Gamma index of 99% in both film planes. The average target TLD agreement was within 1% between the measured and predicted values with the worst TLD agreement within 2%. A subset of the IROC reported results are presented in Figure 5.

## 4 DISCUSSION

Significant care was taken to ensure that the generated beam models were in excellent agreement with measured data. For PDDs, the local DD was evaluated in the 0.5 cm to 29.5 cm depth range, as this was deemed to be most clinically relevant. The measured and calculated PDDs were also visually inspected to ensure agreement outside of this range, shown in Figure 3.

MPPG 5.b. recommends profile comparison in the high dose region should agree within 2% absolute DD, but it does not specify what the high dose region is. For the profile comparison performed, a pass was defined as >90% of the points passing a 2% local DD for a high dose region greater than 80% of the central axis dose. For small fields, such as a 2 × 2 cm^2^ field, the passing criterion was adjusted to require > 90% of points to meet a local 2%/1 mm gamma due to the limited number of measured points (typically 10–20 per profile in the high‐dose region) and the inherent dose gradient of small fields, which are not flat. To evaluate the whole profile including the penumbra region, a global 2%/2 mm gamma was used with a 5% dose threshold. All profiles compared met the passing criterion defined previously and agreed qualitatively in the clinically relevant regions.

Since output factors were measured using both the EDGE and microDiamond detectors, their values were averaged for profile comparison in MCU. MCU scales each profile for absolute dose comparison using the output factor and the dose under reference conditions, therefore, the averaged output factor was applied in MCU for absolute dose comparison. Notably, each individual output factor also met the 2% agreement threshold when compared to EOP.

## 5 CONCLUSION

Excellent agreement was noted for all profile comparisons. All plan measurements passed the gamma criteria recommended in TG‐218 and point dose measurements were within 3% in high‐ and low‐dose regions. The GPUMCD algorithm in EOP has been successfully commissioned and is ready for clinical implementation.

## AUTHOR CONTRIBUTIONS

All authors helped in the development of the manuscript, editing, drafting of the paper, the design of the testing processes, measurements, analysis, review of data, and approved the final version to be published.

## FUNDING INFORMATION

The authors have nothing to report.

## CONFLICT OF INTEREST STATEMENT

Daniel Hyer discloses a consulting relationship with Elekta and research funding from Elekta unrelated to this work. The remaining authors have no conflicts of interest to disclose.
